# Antifungal and Anti-Biofilm Activity of Essential Oil Active Components against *Cryptococcus neoformans* and *Cryptococcus laurentii*

**DOI:** 10.3389/fmicb.2017.02161

**Published:** 2017-11-07

**Authors:** Poonam Kumari, Rutusmita Mishra, Neha Arora, Apurva Chatrath, Rashmi Gangwar, Partha Roy, Ramasare Prasad

**Affiliations:** ^1^Molecular Biology and Proteomics Laboratory, Department of Biotechnology, Indian Institute of Technology, Roorkee, India; ^2^Molecular Endocrinology Laboratory, Department of Biotechnology, Indian Institute of Technology, Roorkee, India; ^3^Molecular Microbiology Laboratory, Department of Biotechnology, Indian Institute of Technology, Roorkee, India

**Keywords:** *Cryptococcus neoformans*, *Cryptococcus laurentii*, biofilm, EO-ACs, SEM, CLSM

## Abstract

Cryptococcosis is an emerging and recalcitrant systemic infection occurring in immunocompromised patients. This invasive fungal infection is difficult to treat due to the ability of *Cryptococcus neoformans and Cryptococcus laurentii* to form biofilms resistant to standard antifungal treatment. The toxicity concern of these drugs has stimulated the search for natural therapeutic alternatives. Essential oil and their active components (EO-ACs) have shown to possess the variety of biological and pharmacological properties. In the present investigation the effect of six (EO-ACs) sourced from Oregano oil (Carvacrol), Cinnamon oil (Cinnamaldehyde), Lemongrass oil (Citral), Clove oil (Eugenol), Peppermint oil (Menthol) and Thyme oil (thymol) against three infectious forms; planktonic cells, biofilm formation and preformed biofilm of *C. neoformans* and *C. laurentii* were evaluated as compared to standard drugs. Data showed that antibiofilm activity of the tested EO-ACs were in the order: thymol>carvacrol>citral>eugenol=cinnamaldehyde>menthol respectively. The three most potent EO-ACs, thymol, carvacrol, and citral showed excellent antibiofilm activity at a much lower concentration against *C. laurentii* in comparison to *C. neoformans* indicating the resistant nature of the latter. Effect of the potent EO-ACs on the biofilm morphology was visualized using scanning electron microscopy (SEM) and confocal laser scanning microscopy (CLSM), which revealed the absence of extracellular polymeric matrix (EPM), reduction in cellular density and alteration in the surface morphology of biofilm cells. Further, to realize the efficacy of the EO-ACs in terms of human safety, cytotoxicity assays and co-culture model were evaluated. Thymol and carvacrol as compared to citral were the most efficient in terms of human safety in keratinocyte- *Cryptococcus* sp. co-culture infection model suggesting that these two can be further exploited as cost-effective and non-toxic anti-cryptococcal drugs.

## Introduction

Cryptococcosis caused by encapsulated basidiomycetes yeast *Cryptococcus* species is an opportunistic fungal infection prominent in the immunocompromised individuals (Martinez and Casadevall, 2015). Among the *Cryptococcus* sp., *Cryptococcus neoformans* remains the major causative agent, however, in the past decade, non-neoformans species such as *Cryptococcus laurentii* and *Cryptococcus albidus* have also been reported to be responsible for 80 percent of the infection (Khawcharoenporn et al., 2007). The need to address the problem of cryptococcosis has significantly increased in past years due to acquired immunodeficiency syndrome (AIDS) epidemic, intensive chemotherapy of cancer patients, solid organ transplant recipients, intravenous drug users and extensive use of immunosuppressive drugs (Kronstad et al., 2011; Shorman et al., 2016). It has been reported that the global burden of cryptococcosis is over one million cases annually, resulting in nearly 625,000 deaths per year (Park et al., 2009). According to United Nations Programme on HIV and AIDS (UNAIDS) report (2016), India has the third largest HIV epidemics (0.26%) in the world with an estimated 68,000 deaths per year. Among the HIV/AIDS patients in Northern India, 3.3% were reported to have cryptococcal infections while in Central India, 3.2% Eucalyptus trees and soil with avian excreta were colonized by *C. neoformans*. Further, from 2005 to 2013, 117 cases of cryptococcosis were reported in Southern India including 87% in HIV positive patients (Berger, 2017).

The ecological strategy that has been associated with such a chronic infection caused by *C. neoformans*, is the formation of biofilm (Ramage and Williams, 2013). A major component of its polysaccharide capsule, glucuronoxylomannan (GXM), plays a central role in biofilm formation and its pathogenesis (Martinez and Casadevall, 2005). The self-produced polysaccharide rich extracellular polymeric matrix (EPM) of biofilm makes the sessile cryptococcal cells resistant to standard antimicrobial therapy resulting in fungal resistance. These biofilm-associated cryptococcal cells are also protected from macrophage phagocytosis in tissues thereby enhancing its quorum sensing and survival (Aslanyan et al., 2017). Some cases of *C. neoformans* and *C. laurentii* forming biofilm have been associated with the ventriculoatrial shunt, indwelling intravascular catheters, cardiac valve and peritoneal dialysis fistula (Ajesh and Sreejith, 2012; Martinez and Casadevall, 2015). This high resistance of biofilm to antifungal drugs compared to their planktonic counterparts is therefore of great clinical relevance (Rizk et al., 2004). For example, *Cryptococcus* sp. biofilm are highly resistant to azole antifungals while amphotericin B and its lipid formulations show good efficacy against biofilm form, however the effective concentrations are above the therapeutic range; thus leading to severe toxicity, renal dysfunction, resulting in the emergence of drug-resistant strains (Brouwer et al., 2004; Delattin et al., 2014). Therefore, it is crucial to develop new drugs and alternative natural therapies that are potentially active against *Cryptococcus* sp. planktonic and its biofilm form.

Phytochemicals including essential oils (EO) and plant extracts isolated from diverse flora have shown to be effective alternatives with a potential to form novel drugs that could effectively be used in the treatment of such kind of recalcitrant infections (Bakkali et al., 2008). The EO and its active components (EO-ACs) have been extensively exploited and described to have antimicrobial, anti-inflammatory and anti-oxidant activities and considered safe in terms of animal and human health usage (Tampieri et al., 2005; Alves-Silva et al., 2016; Darwish et al., 2016). According to a recent report, carvacrol showed antifungal activity against *C. neoformans* strains and thus could be a potent drug component (Nobrega et al., 2016).

The previous studies in this field have majorly focused on the planktonic form, leaving behind a lacuna in the activity of EO-ACs against recalcitrant *Cryptococcus* biofilms. With the objective of filling the said lacuna the present study investigated the effect of essential oil active components (EO-ACs) such as terpenic phenol (thymol, carvacrol, and eugenol); terpenic aldehydes (citral, cinnamaldehyde), and terpenic alcohol (menthol) against *C. neoformans* and *C. laurentii* biofilm formation and preformed biofilms. Moreover, the study pursued to elaborate the effect of the above mentioned terpenic compounds on the cell surfaces and consequent micromorphological changes occurring in the cryptococcal cells. Further, the safety of the effective EO-ACs for the treatment of cutaneous and systemic cryptococcosis was validated by assessing the cytotoxicity of EO-ACs on human keratinocytes and human renal cells along with their efficacy in the co-culture model.

## Materials and methods

### Essential oil active components (EO-ACs) and standard antifungal

The EO-ACs [citral, cinnamaldehyde, menthol, thymol, and eugenol], standard drugs [amphotericin B, nystatin, and fluconazole], were commercially obtained from Sigma-Aldrich, USA. A stock solution of EO-ACs and standard drugs were prepared in dimethylsulfoxide (DMSO, HiMedia, India).

### Fungal strains and growth conditions

Two reference strains *C. neoformans* (NCIM 3541) and *C. laurentii* (NCIM 3373) used in the present investigation, were obtained from National Collection of Industrial Microorganism, Pune. Both the strains were cultured on Sabouraud dextrose agar (SDA, HiMedia, India) for 48 h at 30°C and subcultured monthly. Glycerol stock of the strains was prepared in Sabouraud dextrose broth (SDB, HiMedia, India) and frozen at −80°C. All the experiments were performed in compliance with Biosafety Level 2 (BSL-2) guidelines.

### EO-ACs susceptibility testing

EO-ACs activity against planktonic cells of both the *Cryptococcus* species was performed by standard broth microdilution method recommended by Clinical and Laboratory Standards Institute (CLSI, 2008) reference protocols M27-A3, with a modification of replacing RPMI 1640 medium by Yeast Nitrogen Base (YNB, HiMedia, India). Planktonic cells grown in SDB were harvested at exponential phase, washed with sterile 1X phosphate-buffered saline (PBS pH 7, 0.1M) and re-suspended in YNB medium at a density of 1–5 × 10^4^ cells/mL. Serially double diluted concentration of EO-ACs/drugs (0–1,024 μg/mL) were added in 96-well microtiter plates to provide 0.5–2.5 × 10^4^ cells/mL in 200 μL working volume. YNB medium with 1% DMSO plus 10% mineral oil (HiMedia) was added to control wells (Fontenelle et al., 2007). Tween-80 (HiMedia, India) at 0.05% (v/v) final concentration was added in all assays to enhance EO-ACs solubility. The plates were then incubated at 30°C for 24 h without shaking. After incubation, the growth of cells was measured by microtiter plate reader (SpectraMax, Molecular Devices, USA) at 530 nm and Minimum inhibitory concentration (MIC_80_) which reduces 80% cell growth as compared to control (without EO-ACs/drugs) was determined.

### Biofilm formation and its metabolic activity

Biofilm formation was initiated by culturing *C. neoformans* and *C. laurentii* strains in SDB for 24 h in an incubator shaker at 30°C with 150 rpm. After centrifugation the pellet was washed twice with PBS, followed by counting cells using a haemocytometer, and then suspended at 10^8^ cells/mL in minimal medium (20 mg/mL thiamine, 30 mM glucose, 26 mM glycine, 20 mM MgSO_4_ × 7H_2_O, and 58.8 mM KH_2_PO_4_) (Martinez and Casadevall, 2005). The cell suspension (100 μL) was then added into fetal bovine serum (FBS, Gibco, United States) pre-treated wells of polystyrene 96-well plates (Tarsons, India) and incubated at 30°C without shaking for 2 h for the adhesion of the cells. Biofilms were allowed to form over a series of time intervals (2, 4, 8, 24, 48, and 72 h) with shaking at 70 rpm. After incubation, the wells containing *Cryptococcus* biofilms were washed thrice with 0.05% Tween 20 (HiMedia, India) in PBS to remove non-adherent cryptococcal cells. The cells that still remained attached to the polystyrene surface were considered as true biofilm. All assays were carried out in triplicates.

The biofilm formation was measured using XTT (2,3-Bis-(2-Methoxy-4-Nitro-5-Sulfophenyl)-2H-Tetrazolium-5-Carboxanilide) reduction assay (Martinez and Casadevall, 2005). Filter sterilized stock solution (0.5 g/L) of XTT tetrazolium salt (Sigma-Aldrich, USA) in 1X PBS was stored in aliquots at −80 °C. Preceding assay, an aliquot was thawed and 1 μM freshly prepared menadione (Sigma-Aldrich, Germany) was then added to the XTT solution. The volume of 100 μL of XTT-menadione solution was added into the wells with prewashed biofilm and without biofilm. The plates were then incubated at 37 °C for a period of 4 h in dark. Colorimetric reduction of XTT was measured at 492 nm using microtiter plate reader. The biofilm formation was also assessed using a light microscope (Zeiss, Axiovert 25, Germany).

### Effect of EO-ACs against *Cryptococcus* sp. biofilm formation and preformed biofilms

The biofilm formation assay was performed in 96-well microtiter plates according to the protocol described by Martinez and Casadevall (2006). In brief, the cell suspension was prepared in minimal medium at a density of 2 × 10^8^ cells/mL and dispensed into the wells of microtiter plates. Serially double-diluted concentrations of EO-ACs/drugs (0–1,024 μg/mL) in minimal medium were added to the wells to attain the final cell density of 1 × 10^8^ cells/mL for biofilm formation and plates were incubated at 30°C for 48 h. Subsequently, quantification of biofilms was performed by colorimetric XTT reduction assay and the biofilm inhibiting concentration (BIC_80_), the lowest concentration of EO-ACs/drugs that inhibits 80% metabolic activity of biofilm formation as compared to control (EO-ACs/drugs-free) were determined. For preformed biofilm assay, the biofilm was made as described in previous section (Martinez and Casadevall, 2006). Thereafter, serially double-diluted concentrations of EO-ACs/drugs (0–1,024 μg/mL) were added into the wells of prewashed and preformed biofilms. Minimal medium containing 1% DMSO plus 10% mineral oil without EO-ACs/drugs served as negative control. The microtiter plates were then incubated at 30°C for 48 h, followed by quantification with colorimetric XTT reduction assay and the biofilm-eradicating concentration (BEC_80_), the lowest concentration of EO-ACs/drugs that eradicates 80% of biofilm compared to negative control were calculated.

### Scanning electron microscopy (SEM) and confocal laser scanning microscopy (CLSM) analysis of *C. neoformans* and *C. laurentii* biofilm

The effect of thymol, carvacrol, and citral and the standard drug (amphotericin B) on biofilms were qualitatively analyzed by scanning electron microscopy (SEM) and confocal laser scanning microscopy (CLSM). Biofilms were formed on 20% fetal bovine serum (FBS, Gibco, United States) treated pre-sterilized polystyrene disc (1 mm^2^) in 12-well cell culture plate (Tarsons, India) in the presence of respective BIC_80_ of the above EO-ACs/drug (Martinez and Casadevall, 2007). The cell culture plates were incubated at 30°C for 48 h. Minimum media containing 1% DMSO plus 10% mineral oil without EO-ACs was included as a negative control and amphotericin B treated biofilm served as positive control. At the end of incubation, polystyrene discs were transferred to new 12-well plates and washed thrice with PBS. For SEM, biofilm was washed with PBS and subsequently fixed for 2 h by glutaraldehyde (2.5% v/v) in PBS (0.1M, pH7.5), followed by dehydration in 30, 50, 70, 90, and 100% ethanol solutions. The samples were then dried and sputtered with gold and visualized under scanning electron microscope (Carl Zeiss AG, EVO 40) in high-vacuum mode at 20 kV. Confocal microscopy was performed according to Martinez and Casadevall (2006). For CLSM, *C. neoformans* and *C. laurentii* biofilms were formed in the presence of 32 and 16 μg/mL EO-ACs respectively. Control and treated biofilms were incubated for 45 min at 37°C in 75 μL of PBS containing the fluorescent probes FUN-1 (10 μM, Molecular Probes, USA) and Concanavalin A conjugated to Alexa Fluor 488, (CAAF 488, 25 μM, Molecular Probes, USA). Confocal microscopic examinations of biofilms on polystyrene discs were performed using a Zeiss Axiovert 200 M inverted microscope and the images were analyzed with Zen software.

### Cytotoxicity of EO-ACs in normal human cell lines

Human keratinocyte cell line (HaCaT) and Human embryonic kidney cells (HEK-293) (National Center for Cell Sciences, Pune) were cultured in Dulbecco's modified Eagle's Medium (DMEM, Gibco, United States) supplemented with 10% FBS and 1% antibiotics (Penicillin/streptomycin) solution and kept in a humidified 5% CO_2_ incubator maintained at 37°C. Initially HaCaT and HEK-293 were seeded at a density of 5 × 10^3^ cells/well in 96 well plates. After 24 h of attachment, the cells were incubated in DMEM high glucose media supplemented with and without EO-ACs (thymol, carvacrol, and citral) prepared in DMSO at concentrations (8–256 μg/mL) to check their cytotoxic effect. DMSO (0.1%) served as the negative control. After 24 h incubation at 37°C, MTT [3-(4, 5-Dimethyl-2-thiazolyl)-2, 5-diphenyltetrazolium bromide] assay was performed to measure the cell viability (Mohapatra et al., 2017). MTT (Sigma-Aldrich, USA) was added to the media at a working concentration of 0.5 mg/ml and the cells were incubated in the CO_2_ incubator at 37°C further for 4 h. Then, the media with MTT dye was aspirated from each well and the water insoluble formazan crystals formed were dissolved in 200 μL DMSO directly added to each well of 96 well plates. The plates were placed in a shaker incubator for 30 min for complete dissolution of the formazan crystals. The absorbance value was recorded at 570 nm using Fluostar Optima Plate Reader (BMG Labtech, Germany). The percentage cell viability was estimated by the following formula:

$$\begin{array}{l} \text{Percentage Cell Viability}=(\text{Mean OD of treated cells}\\ \text{ /Mean OD of untreated cells})\;\times 100. \end{array}$$

Subsequently, cytotoxic concentration (CC_50_) at which the cell viability dropped by 50% was recorded.

### Bright-field and fluorescence microscopic analysis of HaCaT and HEK-293

The effect of EO-ACs treatment on the morphology of the normal human cell lines was observed by employing brightfield microscopic imaging. Briefly, 5 × 10^5^ cells/well (HaCaT and HEK293) were seeded in a 12 well plate and incubated for 24 h in a humidified 5% CO_2_ incubator at 37 °C as described in the previous section. The human cells were then treated with the respective MIC_80_ concentration of the EO-ACs against *C. neoformans*. The plates were kept in the incubator further for 24 h. After the treatment period was over, the morphology of the negative control cells and the EO-ACs treated cells were visualized under an inverted light microscope (Zeiss, Axiovert 25, Germany).

The cytotoxic effect and changes in the nuclear morphology due to the administration of EO-ACs were examined with Acridine Orange–Ethidium Bromide (AO-EB) dual staining dye mixture at a concentration 100 μg/mL in PBS (Kasibhatla et al., 2006). The images were captured using 40X objective lens under the fluorescence microscope (Zeiss, Axiovert 25, Germany) with 465 and 563 nm filters respectively.

### Efficacy of EO-ACs in co-culture model of *Cryptococcus* sp. and HaCaT

The co-culture was performed according to a previous study reported by Wong et al. (2014). HaCaT cell culture was performed as described above. The cells were incubated at 37°C in the presence of 5% CO_2_ until confluence was reached with medium changed every day. The cells were washed once with PBS, and fresh medium without antibiotics (as antibiotics could inhibit *Cryptococcus* growth) was added. A cell suspension (1 × 10^4^ CFUs/mL) of *C. neoformans* and *C. laurentii* was prepared in the growth medium DMEM (without antibiotics), and 100 μL of each cell suspension was added into separate wells. Thymol, carvacrol, and citral were added at respective MIC_80_ against *C. neoformans* and *C. laurentii* whereas the untreated vehicle control contained only the growth medium with 0.1% DMSO. The plates were then incubated at 30°C for 24 h in the presence of 5% CO_2_. The viability of HaCaT and cryptococcal cells were subsequently assessed under brightfield and AO-EB dual staining using fluorescence microscopy.

### Statistical analysis

All experiments were performed in triplicate. Data analysis was conducted using SigmaPlot 11.0 (Systat Software, San Jose, CA). The data are expressed as mean ± standard deviations (SD) and statistical significance between treated and control groups was analyzed using One-way Analysis of Variance (ANOVA). Significant difference was defined as *p* < 0.05. IC_50_ values were evaluated using GraphPad Prism.

## Results

### Evaluating the antifungal activity of EO-ACs against planktonic cells of *C. neoformans* and *C. laurentii*

In order to determine the efficacy of EO-ACs (thymol, carvacrol, eugenol, citral, cinnamaldehyde, and menthol) as compared to the standard antifungal drugs (amphotericin B, nystatin, and fluconazole) against *C. neoformans* and *C. laurentii*, the MIC_80_ values were determined. Data showed the planktonic (free-floating) form of *C. neoformans* and *C. laurentii* were more susceptible to polyene drugs (amphotericin B, nystatin) as compared to fluconazole (32 and 16 μg/mL) (Table 1). The MIC_80_ of thymol, carvacrol, and citral against *C. neoformans* were found to be 16, 32, and 64 μg/mL while, *C. laurentii* showed MIC_80_ at 8, 16, and 32 μg/mL respectively (Table 1). Cinnamaldehyde and eugenol showed similar MIC_80_ value (128 μg/mL) against *C. neoformans* while *C. laurentii* was found to be more susceptible to cinnamaldehyde (64 μg/mL) in comparison to eugenol (128 μg/mL). Menthol was the least effective among all the tested EO-ACs against both the species (Table 1).

**Table 1:** List of essential oil active components (EO-ACs) and standard drugs used in the susceptibility study against *Cryptococcus neoformans* and *Cryptococcus laurentii* and their respective MIC_80_, BIC_80_, and BEC_80_ values.

***Cryptococcus* spp**.	***C. neoformans***			***C. laurentii***		
	**MIC_80_**	**${\text{BIC}}_{80}^{\text{a}}$**	**${\text{BEC}}_{80}^{\text{b}}$**	**MIC_80_**	**BIC_80_**	**BEC_80_**
**STANDARD DRUGS (μg/ml)**						
Amphotericin B	1	4	32	0.5	2	16
Nystatin	2	8	64	1	4	64
Fluconazole	32	128	>1,024	16	64	>1,024
**EO-AC (μg/ml)**						
Thymol	16	32	128	8	16	64
Eugenol	128	256	512	128	256	512
Carvacrol	32	64	256	16	32	128
Citral	64	128	256	32	64	256
Cinnamaldehyde	128	256	512	64	128	512
Menthol	256	512	>1,024	128	512	>1,024

*${\text{BIC}}_{80}^{a}$ and ${\text{BEC}}_{80}^{b}$ were determined by measuring XTT reduction activity*.

### Comparison of *C. neoformans* and *C. laurentii* biofilm formation

**Figure 1:**
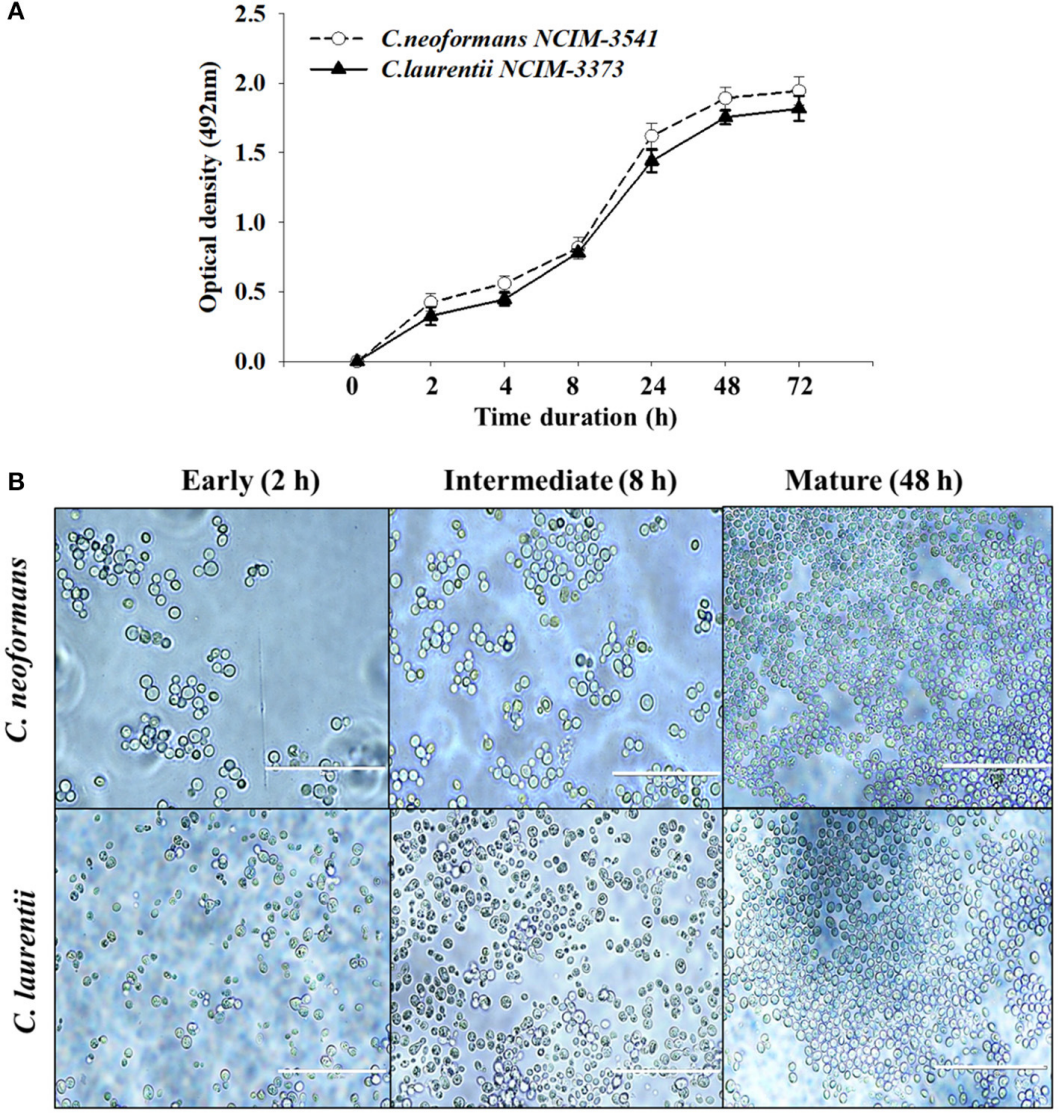
Evaluation of *Cryptococcus* sp. biofilm formation. **(A)** Comparison of kinetics of *Cryptococcus neoformans* (NCIM 3541) and *Cryptococcus laurentii* (NCIM 3373) biofilm formation on polystyrene microtiter plates using colorimetric XTT reduction assay. Error bars represent standard deviation (SD). **(B)** Light microscopic images at different stages of biofilm formation. Images were captured using a 40X power field. Scale bar, 50 μm.

The biofilm formation kinetics was performed up to 72 h time point to optimize and compare the time period for mature biofilm formation for both the fungal species (Figure 1A). Interestingly both *C. neoformans* and *C. laurentii* shared a similar pattern of biofilm growth reaching maturation at 48 h with no significant difference in the metabolic activity rates up to 72 h. However, between these two *Cryptococcus* sp; *C. neoformans* adhered faster as compared to *C. laurentii* during the early stage (2–4 h) in which the cells appeared individual; in a single layer pattern with recurrent budding (Figure 1B). Succeeding, the early phase of adhesion, uniformly distributed micro-colonies of yeast cells throughout the plastic support was observed representing the intermediate stage (8 h). Lastly, the maturation stage was observed at 48 h time point, where the cryptococcal cells were visualized to be in more complex arrangement displaying multi-layered compact structure with increase in the extracellular material surrounding the cell (Figure 1B).

### Determining the effect of EO-ACs against biofilm formation and preformed biofilm

**Figure 2:**
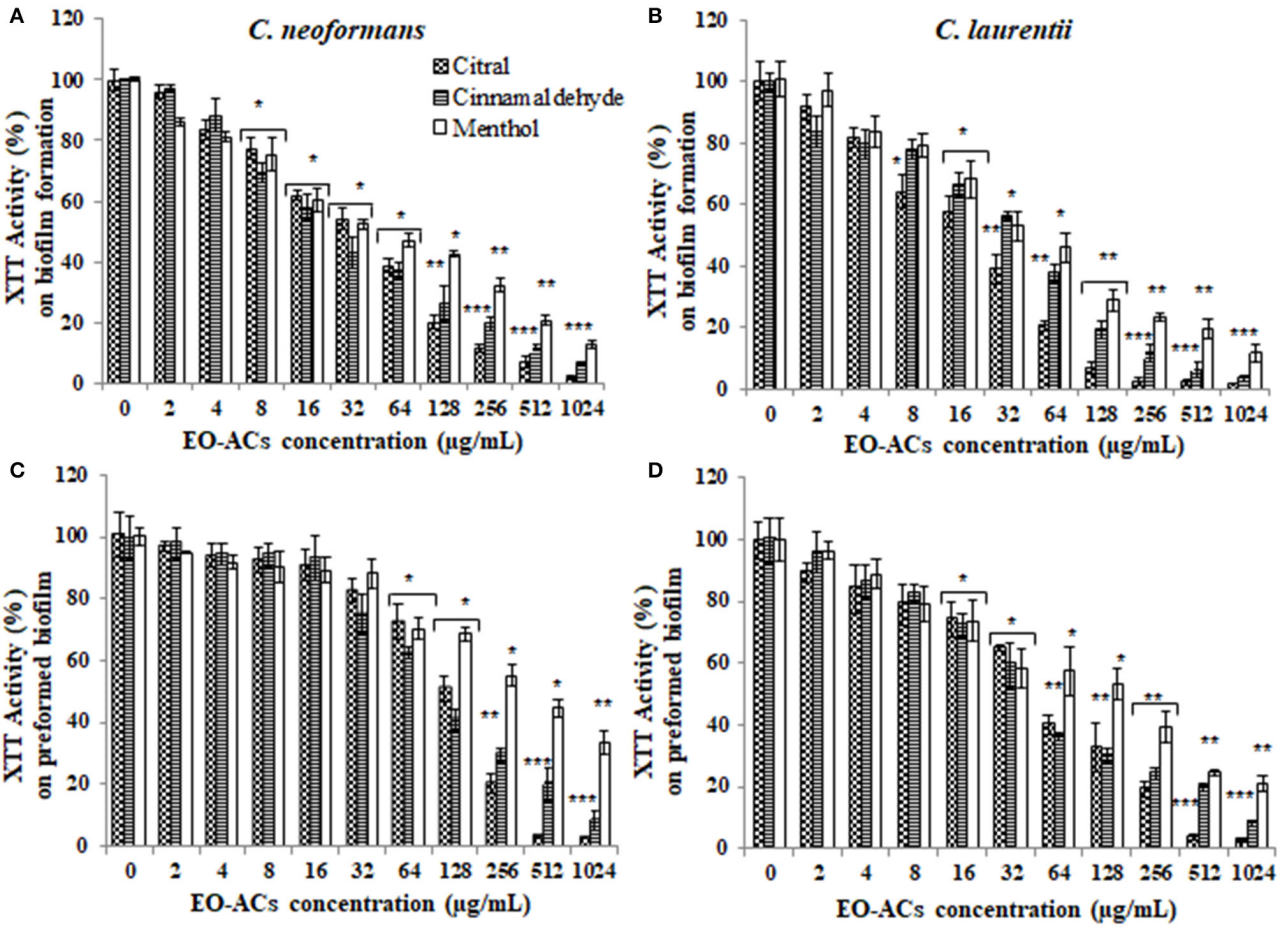
Effect of terpenic aldehyde and alcohol (citral, menthol, and cinnamaldehyde) on *C. neoformans* and *C. laurentii* **(A,B)** biofilm formation **(C,D)** preformed biofilms. Results represent average % metabolic activity ± SD. ^*^p < 0.05, ^**^p < 0.01, ^***^p < 0.001 when compared with control.

**Figure 3:**
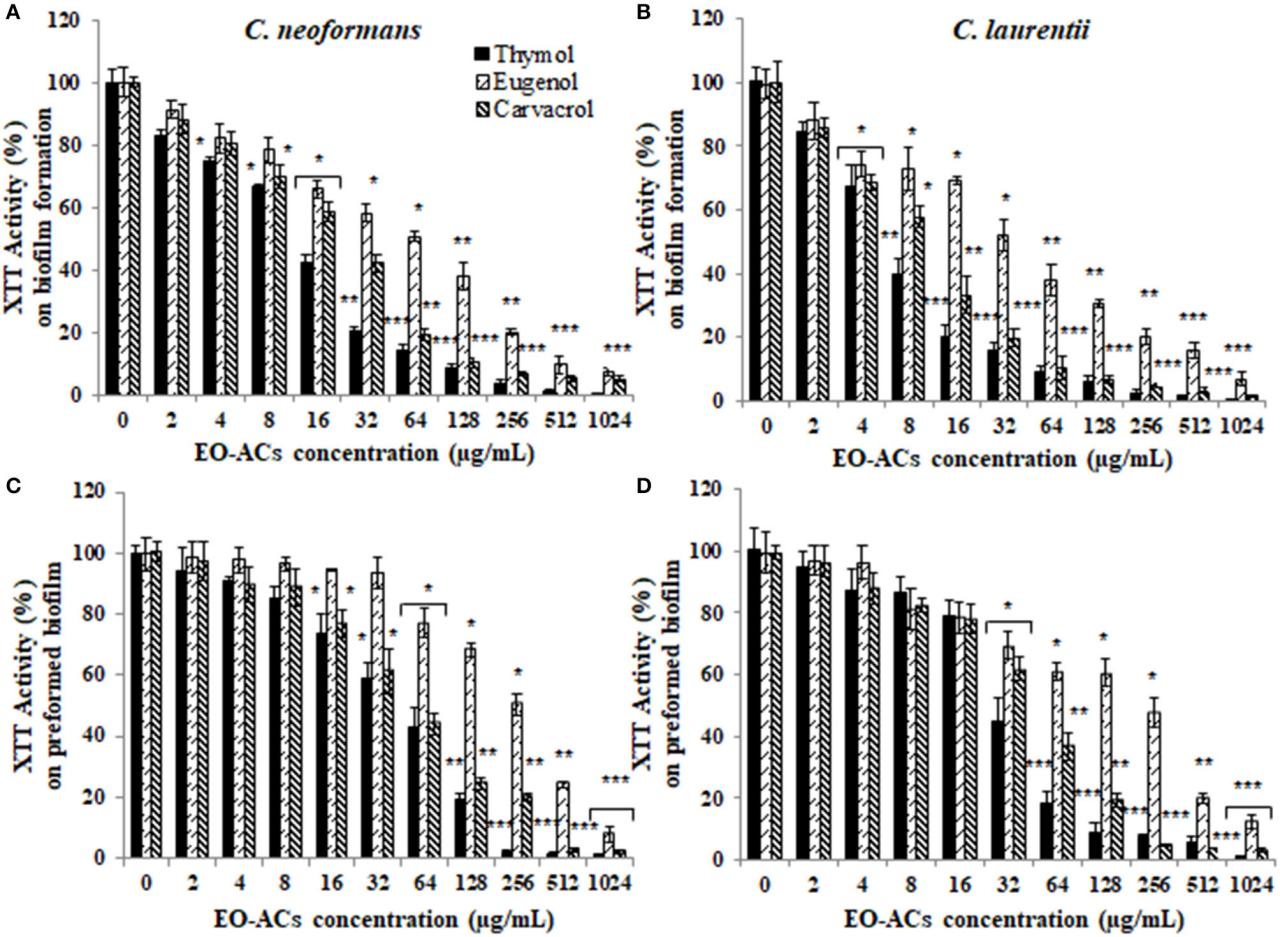
Effect of monohydric phenol (carvacrol, thymol, and eugenol) on *C. neoformans* and *C. laurentii* **(A,B)** biofilm formation **(C,D)** preformed biofilms. Results represent average % metabolic activity ± SD. ^*^p < 0.05, ^**^p < 0.01, ^***^p < 0.001 when compared with control.

The potential and efficacy of EO-ACs against biofilm formation and preformed biofilm was determined in terms of BIC_80_ and BEC_80_ respectively and the viability was expressed as percentage metabolic activity. The BIC_80_ of amphotericin B (4, 2 μg/mL); nystatin (2 μg/mL) and fluconazole (128, 64 μg/mL) against *C. neoformans* and *C. laurentii* were 4-fold higher than their planktonic MICs, demonstrating that biofilm-associated cryptococcal cells are substantially more resistant to drugs than their planktonic counterparts (Table 1). Among the EO-ACs, menthol showed the maximum BIC_80_ (512 μg/mL) against both the *Cryptococcus* species (Figures 2A,B) while, thymol exhibited minimum BIC_80_ of 32 and 16 μg/mL respectively which was comparable with the standard drugs (Figures 3A,B). Eugenol prevented the biofilm formation at 256 μg/mL, which was twice the MIC. Further, it was observed that effective concentration of citral and carvacrol against biofilm formation of *C. laurentii* was observed to be up to 50% less as compared to the potency of the same against *C. neoformans* biofilm formation (Table 1).

Moreover, on evaluating the efficacy of EO-ACs and standard drugs on preformed biofilms, the data showed that the polyene drugs inhibited the preformed biofilm at the concentration 16-fold higher than their respective BIC_80_ while fluconazole failed to eradicate biofilm even at the highest concentration tested demonstrating preformed biofilm resistance (Table 1). On the other hand, the BEC_80_ value of 256 and 512 μg/mL was recorded in biofilm treated with citral and cinnamaldehyde respectively. Menthol showed the minimum anti-biofilm activity and was unable to eradicate the biofilm even at the highest concentration of 1,024 μg/mL (Figures 2C,D). Furthermore, BEC_80_ (128 and 256 μg/mL) of thymol and carvacrol against *C. neoformans* were 4-fold and 8-fold higher as compared to their biofilm forming planktonic cells and free-floating planktonic cells respectively (Figure 3C). The BEC_80_ (64 and 128 μg/mL) of thymol and carvacrol against *C. laurentii* was comparatively lower (Figure 3D). Citral was equally effective in eradicating preformed biofilms of both the species with BEC_80_ of 256 μg/mL.

Overall these results suggest that the six tested EO-ACs exhibits anti-biofilm activity against *C. neoformans* and *C. laurentii* in the following order: thymol>carvacrol>citral>eugenol = cinnamaldehyde>menthol respectively.

### Analyzing the changes in biofilm cells morphology after EO-ACs treatment

Based on the above BIC_80_ and BEC_80_ results, most potent EO-ACs (Citral>Carvacrol>Thymol) were selected for visualizing the changes in the cell morphology of cryptococcal cells using SEM and Confocal Imaging. The scanning electron micrographs showed that the control cells at 0 h had smooth outer cell surface (Figures 5A,G) and untreated biofilm (48 h) exhibited agglomeration of cryptococcal cells with EPM (as indicated by the black arrows) (Figures 5B,H). However, on treatment of *C. neoformans* and *C. laurentii* biofilms at BIC_80_ of citral (128 and 64 μg/mL), no visible EPM was detected along with significant reduction in the density of cells (Figures 5D,J). Citral also caused aberrations in the cell membrane resulting in the bursting of cells. Similarly, biofilm treated in the presence of BIC_80_ of carvacrol (64 and 32 μg/mL) showed disruption with irregular cell surface and oozing out of cellular content (Figures 5E,K). Further, treatment of *C. neoformans* biofilm to BIC_80_ thymol against both the species, the biofilm formation was almost completely inhibited with only a few cells left. The shrinkage of the cells was also noticed (Figures 5F,L). In general, the SEM images of morphological alterations in cryptococcal cells in the presence of EO-ACs were comparable to those of the positive control group (amphotericin B, BIC_80_) suggesting the efficacy of the above EO-ACs in inhibiting the growth of *Cryptococcus* sp. biofilms.

**Figure 4:**
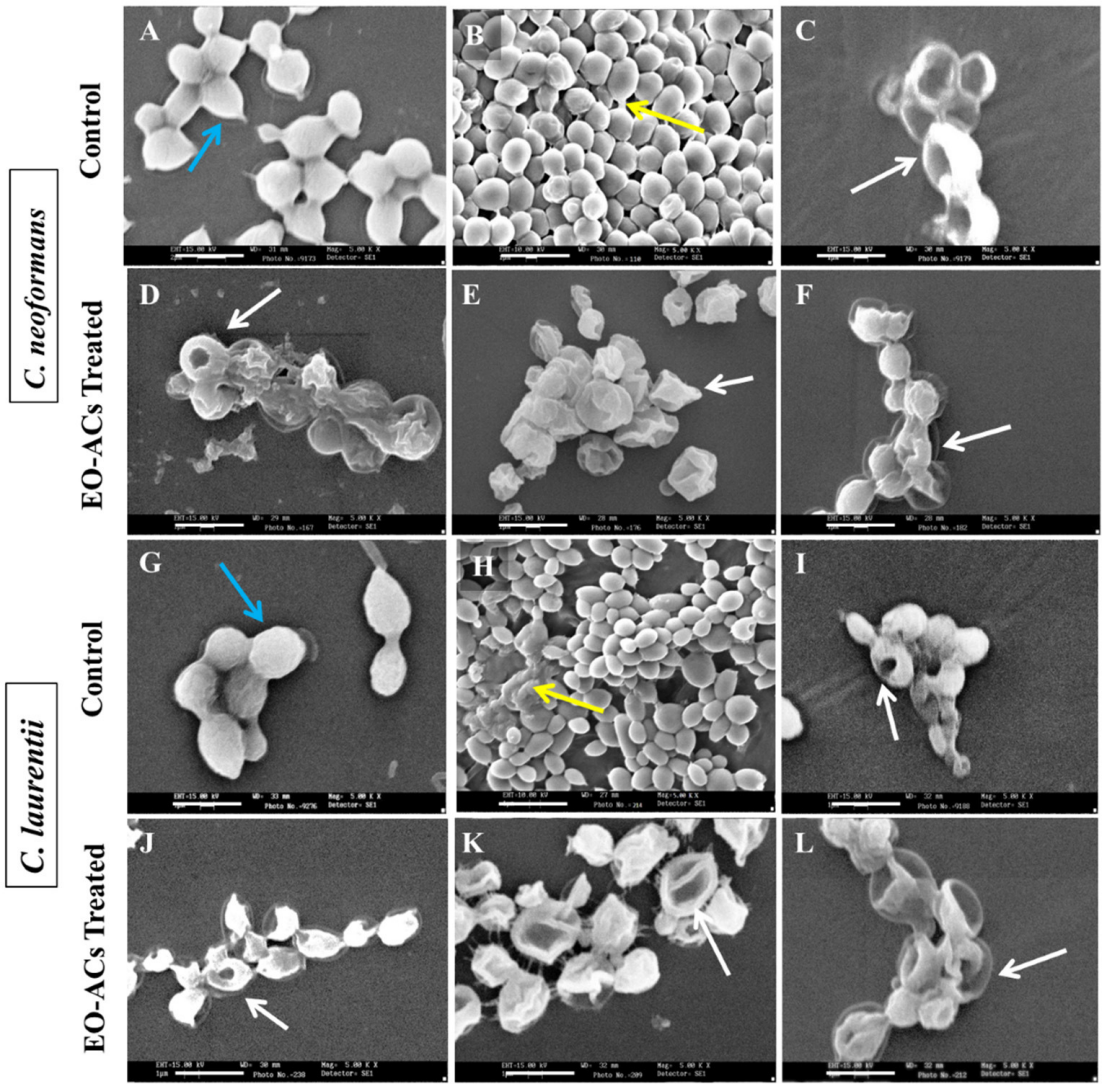
Scanning electron microscopic images of *C. neoformans and C. laurentii* biofilm formed in the absence and presence of EO-ACs on polystyrene disc in 12-well culture plates at 30 °C for 48 h. **(A,G)** Negative control: 1% DMSO + 10% mineral oil (MO) at 0 h **(B,H)** biofilms after 48 h **(C,I)** Positive control: amphotericin B (4 and 2 μg/mL) **(D,J)** EO-ACs treatment: citral (128 and 64 μg/mL) **(E,K)** carvacrol (64 and 32 μg/mL) and **(F,L)** thymol (32 and 16 μg/mL). In the control panel; Blue arrows indicate normal cells with a smooth surface and yellow arrows show extracellular polymeric matrix (EPM) of biofilm, while in treatment panel; white arrows indicate structural and morphological changes after treatment. Magnification 5000X, bar 1 μm.

**Figure 5:**
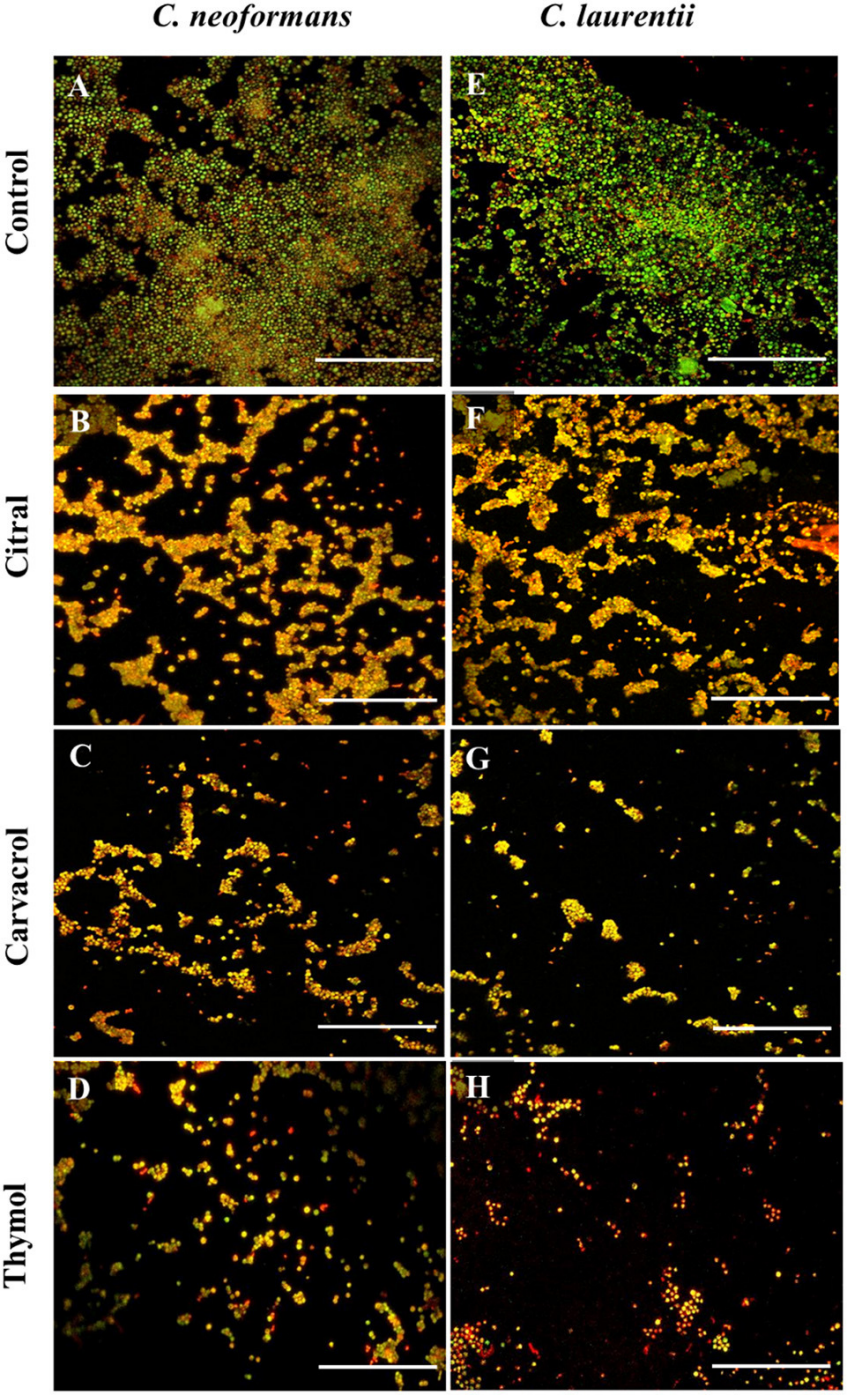
Confocal laser scanning microscopic images of *C. neoformans and C. laurentii* biofilms formed before and after treatment with EO-ACs. **(A,E)** Control (1% DMSO) **(B–D)** 32 μg/ml of citral, carvacrol and thymol **(F–H)** 16 μg/ml of citral, carvacrol and thymol. In the control panels, images of biofilm showed metabolically active (red, FUN-1-stained) cells embedded in the EPM (green, CAAF- 488 -stained). In the citral, carvacrol and thymol treatment panels, the yellow-brownish region represents metabolically non-viable cells. The images were taken by using 40X power field. Scale bar 50 μm.

The visual effects on the biofilm structure and morphology were analyzed using FUN-1 and CAAF 488. In metabolically active cells, FUN-1 (excitation wavelength = 470 nm; emission = 590 nm) get converted into red cylindrical intravacuolar structure. CAAF 488 (excitation wavelength = 488 nm; emission = 505 nm) binds to glucose and mannose residues of the cell wall and capsule polysaccharides (EPM) and fluorescence green (Martinez and Casadevall, 2006). Regions of red fluorescence (FUN-1) correspond to live or viable cells while, the green fluorescence (CAAF-488) indicates cell wall or capsule polysaccharides, and yellow-brownish areas signify metabolically inactive or non-viable cells. The confocal micrographs of the mature *C. neoformans* biofilm (control) displayed a composite structure with a nexus of viable cells emitting red fluorescence along with EPM giving out green fluorescence (Figure 5A). On the other hand, biofilms treated with 32 μg/mL of citral was marked by a decrease in EPM and metabolic activity of cells as some area appeared yellowish brown, while carvacrol treated biofilm at the same concentration showed less number of cells and thymol treated cells showed nearly complete inhibition of biofilm with dead yeast cells (Figures 5B–D). The same pattern was also observed in case of *C. laurentii* biofilm but at a lower concentration of 16 μg/mL (Figures 5F–H).

### Assessing the cytotoxicity of EO-ACs in normal human cell lines

**Figure 6:**
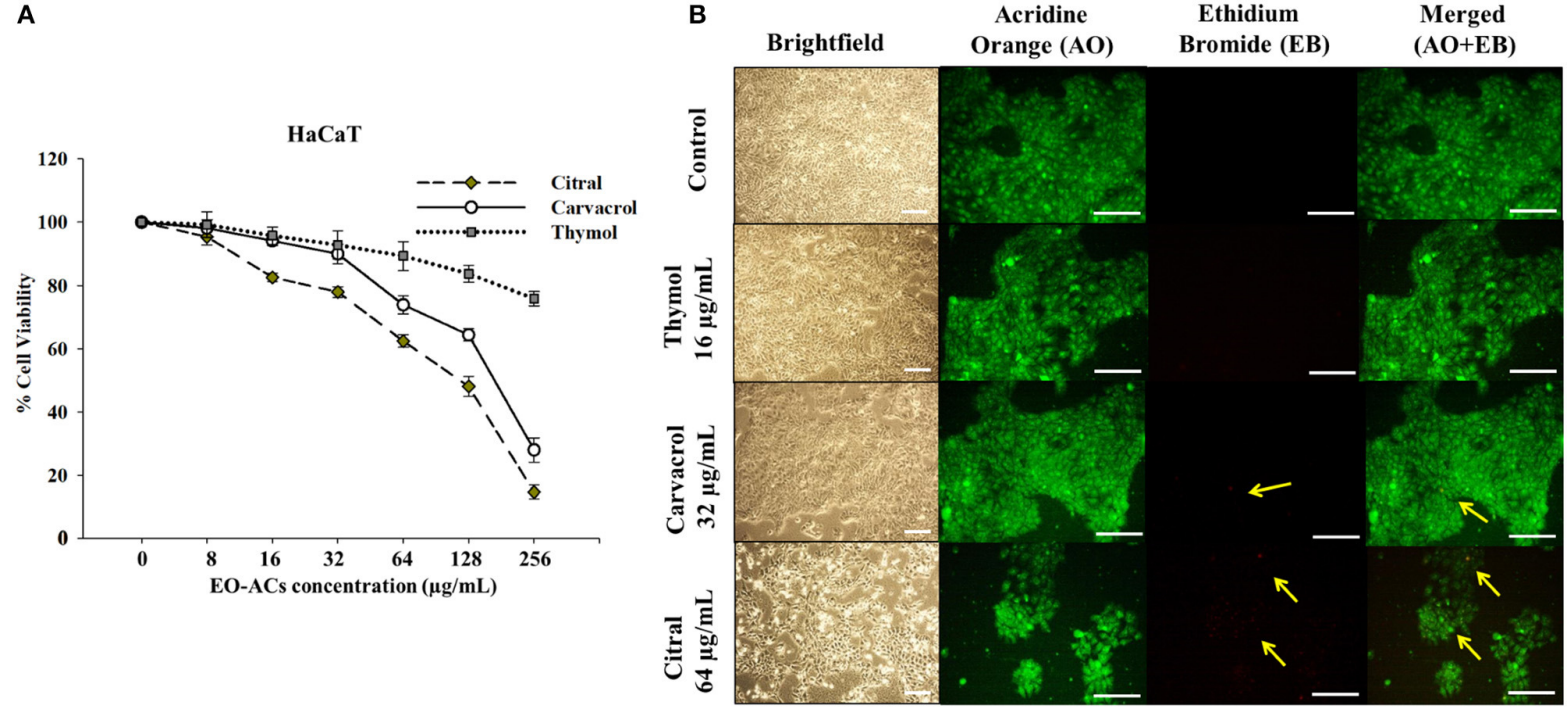
Cytotoxic effects of EO-ACs on HaCaT. **(A)** The cytotoxic effect of thymol, carvacrol, and citral on normal human keratinocytes viability **(B)** Brightfield and fluorescence microscopy of HaCaT cells before and after treatment with thymol, carvacrol, and citral at their respective MIC_80_ Control and treatment panels show fluorescence images stained with acridine orange and ethidium bromide for live/dead cells (Green: live cells and Red: dead cells). Yellow arrows indicate dead keratinocytes. The brightfield images were taken using 10X power field and fluorescence images were taken using 40X power field. Scale bar 50 μm.

**Figure 7:**
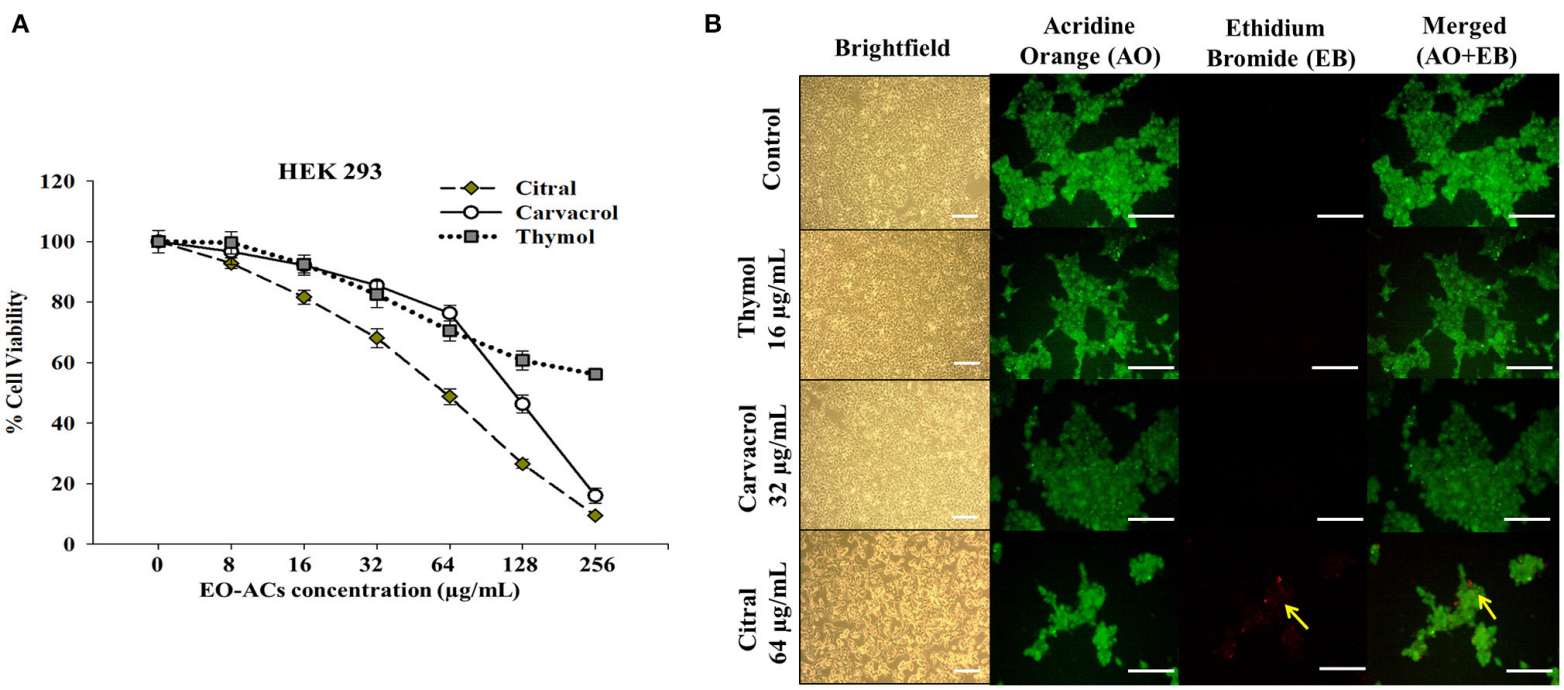
Cytotoxic effects of EO-ACs on HEK-293. **(A)** The cytotoxic effect of thymol, carvacrol, and citral on normal human renal cells viability **(B)** Brightfield and fluorescence microscopy of HEK-293 cells before and after treatment with thymol, carvacrol, and citral at their respective MIC80. Control and treatment panels show fluorescence images stained with acridine orange (AO) and ethidium bromide (EB) for live/dead cells (Green: live cells and Red: dead cells). Yellow arrows indicate dead renal cells. The brightfield images were taken using 10X objective lens and fluorescence images were taken using a 40X objective lens. Scale bar 50 μm.

*C. neoformans* and *C. laurentii* causes disseminated cryptococcosis affecting organs like skin and kidney (ref). Therefore, in order to establish the non-toxicity of the above EO-ACs (thymol, carvacrol, and citral) on the normal human cell line, the cytotoxic activity was tested in HaCaT and HEK-293. The EO-ACs decreased cell viability in a concentration-dependent manner on both the cell lines. The results revealed that CC_50_ of thymol was 1,280 ± 3.11 μg/mL and 283.65 ± 2.45 μg/mL in HaCaT and HEK 293 respectively (Table 2). Further, treatment with thymol at 16 μg/mL reduced the viability of HEK-293 cells by 7.5% compared with the untreated controls (untreated), which was low as compared to citral (18.4% reduction) (Figure 7A). Thymol and citral at 64 μg/mL caused 10.7 and 37.6% reduction in HaCaT cell viability compared to the controls, respectively (Figure 6A). However, carvacrol treatment reduced the viability of HaCaT and HEK-293 by 10.1 and 14.5% respectively as compared to the treatment free controls (Figures 6A, 7A). The results suggested that among the above EO-ACs; thymol exhibited the minimum cytotoxicity while citral showed the maximum cytotoxic activity against both the human cell lines.

**Table 2:** Evaluation of cytotoxic concentration of thymol, carvacrol, and citral on normal HaCaT and HEK-293 cells.

**EO-ACs Treatment**	**Cytotoxicity CC_50_ (μg/mL)**	
	**HaCaT**	**HEK-293**
Thymol	1,280.00 ± 3.11	283.65 ± 2.45
Carvacrol	154.82 ± 2.16	113.00 ± 2.05
Citral	93.20 ± 1.96	57.41 ± 1.75

*The CC_50_ values are mean ± SD of three independent experiments (p < 0.05)*.

### Bright-field and fluorescence microscopic analysis of HEK-293 and HaCaT

The bright field images of HEK293 cells and HaCaT cells after treatment at respective MIC_80_ of the three EO-ACs for 24 h, showed no morphological alteration in response to the thymol and carvacrol treatment whereas little morphological disruption were visible in the citral treated cells (Figures 6B, 7B). These results were well supported by the fluorescence micrographs of HEK-293 and HaCaT cells labeled by fluorescent probes AO/EB. The AO dye is permeable to both live and dead cells and stains all nucleated cells to generate green fluorescence while the EB stains cells that have lost membrane integrity and thus emit red fluorescence (Mohapatra et al., 2017). In combination, apoptotic cells stain yellowish orange and necrotic cells stain reddish orange. Dual staining confirmed that the control group, thymol, and carvacrol treated groups showed the maximum number of AO stained viable human cells with normal cellular morphology whereas citral treated cells showed some EB stained cells as an indication of apoptosis/cytotoxicity (Figures 6B, 7B).

### Evaluating the efficacy of EO-ACs in co-culture model of *Cryptococcus* sp. and HaCaT

**Figure 8:**
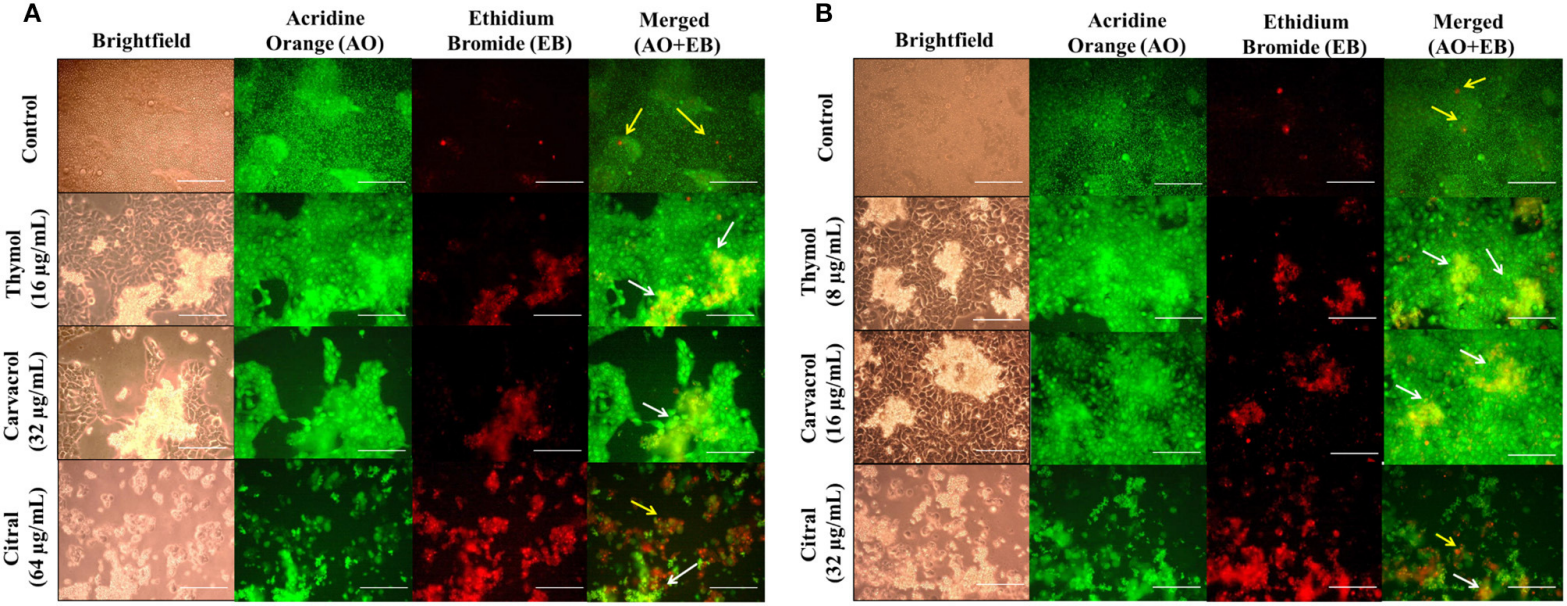
Effect of EO-ACs (thymol, carvacrol, and citral) treatment in Cryptococcus-HaCaT coculture model **(A)** *C. neoformans* **(B)** *C. laurentii*. The viability of co-cultured HaCaT and cryptococcal cells in the presence of EO-ACs was assessed by fluorescence microscopy using AO-EB dual staining. Control panel shows uniformly dispersed live cryptococcal cells (green, AO stained) with few dead HaCaT cells (red, EB stained). Thymol and carvacrol treatment panel show live HaCaT (green) and sparse and unevenly distributed dead cryptococcal cells (red). Citral treatment panels show dead cryptococcal cell with necrotic HaCaT cells. Yellow arrows indicate dead keratinocytes and the white arrow indicate dead cryptococcal cells. The brightfield and fluorescence images were taken using a 40X objective lens. Scale bar 50 μm.

The EO-ACs efficacy at their respective MIC_80_ values to prevent cutaneous cryptococcosis was evaluated using a co-culture model of human keratinocytes; HaCaT infected with *C. neoformans* and *C. laurentii*. The co-culture model was also helpful in visualizing a real model of cryptococcal cells coexisting with keratinocytes incubated with the tested EO-ACs. The brightfield micrographs showed vehicle control (0.1% DMSO) with uniformly dispersed cryptococcal cells along with HaCaT cells (Figures 8A,B). The corresponding fluorescence micrographs allowed for a qualitative assessment of the distribution of live cryptococcal cells (green color) with respect to live (green color) and dead (red color) keratinocytes (Figures 8A,B). Co-culture model treated with EO-ACs showed sparse and uneven accumulation of *C. neoformans and C. laurentii* (red color) compared to the control. Thymol and carvacrol treated cryptococcal cells were observed to be dead and stained red with EB while, most of the HaCaT cells stained green with AO indicating the specific action of the EO-ACs toward *C. neoformans* and *C. laurentii*. However, at MIC_80_ of citral, apoptosis of both, the cryptococcal cells and some of the HaCaT cells were observed.

## Discussion

In recent years, a major concern associated with managing cryptococcosis is the emergence of antimicrobial resistant strains of *C. neoformans* and *C. laurentii* along with their competence to form recalcitrant biofilms in medical settings (Ajesh and Sreejith, 2012; Smith et al., 2015). The ability of fungi to develop such resistance against a drug is an evolutionary process and cannot be abrogated (Srinivasan et al., 2014). Therefore, it has become indispensable to identify/develop a novel class of drugs that are natural and directed against targets which do not impart selective pressure or promote drug-resistance. In this regard, EO-ACs offers a great potential for developing novel broad-spectrum key molecules against a wide range of drug-resistant pathogenic microbes (Swamy et al., 2016). Recently, Cardoso et al. (2016) and Cavaleiro et al. (2015) reported strong antifungal activity of EO-ACs of *Ocimum basilicum* (linalool and geraniol) and *Angelica major* (α-pinene and cis-β-ocimene) against *C. neoformans* and anti-biofilm activity against *Candida* species. Keeping this in view, the present study evaluated the holistic efficacy of six EO-ACs (thymol, carvacrol, eugenol, citral, cinnamaldehyde and menthol) against *Cryptococcus* sp. three infectious forms, i.e., planktonic, biofilm formation and preformed biofilm respectively.

Among the tested EO-ACs; thymol, carvacrol, and citral were selected based on criteria proposed by Morales et al. (2008) for an antimicrobial potential of products which considers a product with MIC: <100 μg/mL to have strong antimicrobial activity. Thymol was the most effective EO-ACs which efficiently inhibited *Cryptococcus* sp.; planktonic cells, biofilm formation and mature biofilms (Table 1, Figure 3). Its activity was closely followed by that of carvacrol and citral (Figures 2, 3). Even though, the standard drugs (amphotericin B, nystatin) used in the present study inhibited the planktonic form of the cryptococcal cells at a much lower concentration (1 and 2 μg/mL) than the EO-ACs, however when evaluated for their efficacy against biofilm forms, the BEC_80_ values were 32–64 times higher than its MIC_80_ values. On the other hand, the BEC_80_ of thymol, carvacrol, and citral against preformed biofilm were only 4–8-fold higher than planktonic cells (Table 1). These results suggested that the biofilms of *C. neoformans* and *C. laurentii* were significantly more susceptible to the terpenic compounds in comparison to the standard drugs. The study also revealed that the biofilm of *C. laurentii* was more susceptible to the EO-ACs in comparison to *C. neoformans*, which was in line with a similar study on biofilms of *Candida albicans* and non-albicans species demonstrating the presence of species-specific and drug-specific differences respectively (Simitsopoulou et al., 2013).

Such antifungal and anti-biofilm activity of thymol (monoterpenoid found in the thyme oil) can be attributed to its structure 2-isopropyl-5-methylphenol (Borugă et al., 2014). The *in vitro* findings of the study was confirmed with the SEM and confocal images of cells treated with thymol which showed a significant reduction in the number of cells with deformed and perforated outer membrane (Figures 4, 5). These morpho-structural alterations of cells and its damage are more likely due to penetration of thymol into cell membrane outer layer resulting in expansion of dipalmitoyl-phosphatidylcholine (DPPC) monolayers. This causes a decrease in surface elasticity and thereby changing the lipid bilayer morphology which leads to extreme rapid efflux of intracellular constituents (Ferreira et al., 2016).

Carvacrol [2-Methyl-5-(propan-2-yl) phenol] is found alone or in combination with its isomer thymol in oregano and thyme oil (Nostro et al., 2007). The effective concentration of carvacrol against planktonic and biofilm forms of *Cryptococcus* sp. was 2-fold higher than thymol (Table 1). Various different mechanism of action have been proposed for carvacrol which includes Ca^2+^ stress and inhibition of the TOR (Target of Rapamycin) pathway resulting in the formation of lesions on the membrane thereby blocking the ergosterol biosynthesis leading to the damage of enzymatic cell systems involved in energy production and synthesis of structural compounds (Suntres et al., 2015). Recently Chaillot et al. (2015) reported that carvacrol acts as an antifungal agent by changing endoplasmic reticulum (ER) integrity causing ER stress and induction of the unfolded protein response (UPR). All these modes in synergy or individually can affect the membrane integrity of the fungal cell(s).

Lastly, citral (3,7-dimethyl-2-6-octadienal) is a mixture of geranial (citral A) and neral (citral B) which naturally occurs in lemongrass oil (Silva et al., 2008). Few authors have previously reported the antifungal activity of citral against planktonic cells of *C. neoformans* (Viollon and Chaumont, 1994; Lima et al., 2005). The present study displayed that the concentration of citral required to inhibit biofilm formation (BIC_80_) and eradicate preformed biofilm (BEC_80_) was 2 and 4-fold higher compared to carvacrol and thymol respectively (Figures 2, 3). The morphological observation in case of citral against *Cryptococcus* sp. was supported by a similar study reporting the action of lemongrass oil rich in citral against *C. albicans* which caused deleterious changes in cell surface and structure (Tyagi and Malik, 2010). Citral mainly targets fungal cell membrane by blocking its synthesis and affecting membrane structure by inhibiting spore germination, proliferation and cellular respiration (Harris, 2002). However, its mechanism of action does not involve cell wall or ergosterol (Leite et al., 2014). Considering the fact that immunocompromised individuals are the most vulnerable host to a number of pathogenic infection. The present study was compared with the previous studies carried out on pathogenic fungi (*Candida* sp.) and other bacterial species (*Staphylococcus* sp., *Klebsiella* sp., *Pseudomonas* sp., *Acetinobacter* sp.) (Table 3). The comparison showed the broad spectrum potential of thymol, carvacrol, and citral against the above listed biofilm forming micro-organisms, illustrating the significance of using these EO-ACs for developing universal drug therapies.

**Table 3:** Comparative analysis of antimicrobial and anti-biofilm potential of thymol, carvacrol and citral against biofilm forming micro-organisms.

**MicroOrganisms (Strain)**	**Essential Oil Active Components (EO-ACs) (μg/ml)**									
	**Thymol**			**Carvacrol**			**Citral**			**References**
	**MIC**	**BIC**	**BEC**	**MIC**	**BIC**	**BEC**	**MIC**	**BIC**	**BEC**	
*Stapylococcus aureus* (6ME^a^, ATCC 6538^b^)	300^a^	600^a^	2,500^a^	100^a^	600^a^	2,500^a^	500^b^	500^b^	1,000^b^	Nostro et al., 2007
*Stapylococcus epidermidis* (ATCC 35984)	600	1,250	5,000	300	1,250	5,000	-	-	-	Nostro et al., 2007
*Klebsiella pneumoniae* (OXA-48)	200	200	-	125	125	-	-	-	-	Raei et al., 2017
*Pseudomonas aeruginosa* (GIM)	800	800	-	250	500	-	-	-	-	Raei et al., 2017
*Acetinobacter baumanni* (SIM)	200	400	-	62	250	-	-	-	-	Raei et al., 2017
*Candida albicans* (ATCC 66396^d^, 04^d^)	100^c^	-	300^c^	97.6^c^	-	292.8^c^	148.62^d^	-	594^d^	Dalleau et al., 2008
*Candida glabrata* (IHEM 9556^e^, ATCC 2001^f^)	200^e^	-	1,250^e^	195.2^e^	-	1,250^e^	128^f^	-	-	Dalleau et al., 2008
*Candida parapsilosis* (ATCC 22019)	200	-	600	195.2	-	1,250	500	-	5,000	Dalleau et al., 2008
*Cryptococcus neoformans* (NCIM 3541)	16	32	128	32	64	256	64	128	256	This study
*Cryptococcus laurentii* (NCIM 3373)	8	16	64	16	32	128	32	64	256	This study

Data values

a, b, c, d, e, f *corresponds to the tested strains; - not tested on the strain*.

However, to fully realize the potency of thymol, carvacrol, and citral, the study also evaluated their toxicity against the human cell lines HaCaT and HEK-293 using *in vitro* model. The fungal and humans cells are eukaryotic and being similar in nature poses a major hurdle in antifungal development. Thus, fungus-specific drug targets to avoid undesirable cytotoxicity to human cells are required (Krcmery and Kalavsky, 2007). Data showed that the CC_50_ of thymol and carvacrol for HaCaT was 80–160 and 10–20-folds higher while for HEK-293 it was 18–35 and 7–14-folds higher than its MIC_80_ against *C. neoformans* and *C. laurentii* respectively. This suggested that there is a range of concentrations at which these two EO-ACs could be used as an antifungal agent without causing significant toxicity to human cells (Table 2). However, the CC_50_ (57.4 and 93.2 μg/mL) of citral against the above two cell lines was comparable to its MIC_80_ rendering it cytotoxic to humans. The above findings corroborated with a previous study that proves thymol and carvacrol as the safe option while do not recommend citral as potential therapeutic agent (Elshafie et al., 2017). Additionally, the above results were supported by fluorescence microscopy of the co-culture model that showed a significant decrease in viable cryptococcal cells dispersed among keratinocytes in the presence of EO-ACs (Figures 8A,B). It is important to note that there were no morphological changes in the thymol and carvacrol treated HaCaT cells in comparison to the control cells. Further, the overall density and the distribution of live keratinocytes (green color) appeared similar to the control. However, citral treatment though killed cryptococcal cells but also resulted in a loss of 62.4% cell viability of keratinocytes, thereby making it unsafe for human usage (Figure 6A). Moreover, both carvacrol and thymol have been classified as GRAS (generally recognized as safe) and their use in food has been approved by European Parliament and Council (Hyldgaard et al., 2012) making them a potential option for developing anti-cryptococcal drugs.

## Conclusion

To conclude, this is the first study exploring efficacy and safety of three EO-ACs; thymol, carvacrol, and thymol against *C. neoformans* and *C. laurentii*. The obtained results corroborated that thymol and carvacrol could be promising, efficient and cost-effective drugs for the inhibition of *Cryptococcus* biofilms. However, it is worthy to note that standard drugs showed antifungal and anti-biofilm activity at a lower concentration as compared to above EO-ACs but these concentrations are much beyond the therapeutic range causing severe toxicity. Hence future studies may investigate the efficacy of combinational therapy of EO-ACs with standard drugs or with other EO-ACs which could lead to novel drug therapies against recalcitrant infections.

## Author contributions

PK and RP conceived and designed the experiments. AC and RG along with PK performed antimicrobial work. RM under the supervision of PR carried out cell culture work. PK analyzed the data and wrote the manuscript. RP and NA gave critical revisions of intellectual content along with final approval for publication.

### Conflict of interest statement

The authors declare that the research was conducted in the absence of any commercial or financial relationships that could be construed as a potential conflict of interest.
